# Anthropogenic noise exposure suppresses the immune response in *Mytilus* spp. following *Vibrio* sp*lendidus* challenge

**DOI:** 10.3389/fimmu.2025.1657667

**Published:** 2025-09-18

**Authors:** Ambre F. Chapuis, Matthew A. Wale, Morgan Bailey, Hannah M. Farley, Tim P. Bean, Tim Regan

**Affiliations:** ^1^ The Roslin Institute and Royal (Dick) School of Veterinary Studies, The University of Edinburgh, Midlothian, United Kingdom; ^2^ Centre for Conservation and Restoration Science, School of Applied Sciences, Edinburgh Napier University, Edinburgh, United Kingdom

**Keywords:** mussel (*Mytilus spp.*), noise - exposure, stress response, transcriptomics (RNA sequencing), *Vibrio splendidus*, GFP tagging

## Abstract

**Introduction:**

Anthropogenic noise is a growing environmental stressor in marine ecosystems, yet its effects on immune function in bivalves remain poorly understood.

**Methods:**

This study examined the transcriptional response of blue mussels, *Mytilus* spp., following exposure to ship noise for seven days, followed by a low-dose *Vibrio splendidus* bath challenge.

**Results:**

Transcriptomic analysis at multiple time points postnoise exposure revealed only subtle changes in expression signatures which appeared to resolve at later time points. However, compared with the controls, mussels exposed to ship noise showed a reduced number of differentially expressed genes in their gill tissue following bacterial challenge. This indicated a suppressed immune response, as indicated by reduced expression of immunerelated genes compared to controls. While bacterial burden and mortality did not significantly differ between noise-exposed and control groups, the proportion of GFP-tagged *Vibrio splendidus* colonies was higher in noise-exposed mussels.

**Conclusions:**

These findings contribute to a growing body of evidence that anthropogenic noise may impair immune function in bivalves, with implications for aquaculture and marine ecosystem health.

## Introduction

Anthropogenic noise pollution is increasingly recognised as a significant environmental stressor in marine ecosystems. Increasing levels of underwater noise, driven by vessel traffic, industrial activity, and offshore construction have led to elevated ambient noise levels that can impact marine organisms at multiple biological scales from behaviour to physiology and immune function (1–3). While much research has focused on noise impacts in fish and marine mammals, invertebrates, including *Mytilus* spp. (blue mussels), are also affected (1), yet remain comparatively understudied.

*Mytilus* spp. play critical ecological and economic roles. These reef-building bivalves are ecosystem engineers, dominating fouling communities and forming dense aggregations on hard substrata in shallow coastal zones (4). Their filter-feeding activity reduces eutrophication and mediates nutrient cycling, with potential for bioremediation applications, including pollution monitoring and reducing environmental impacts of aquaculture waste from e.g. salmon farms (5–7).

As a key aquaculture species, *Mytilus* spp. underpin significant shellfish production globally (8), and are the second most heavily produced aquaculture species in the UK (9). However, mussel populations face increasing threats from environmental stressors, including warming seas, ocean acidification, and emerging pathogens (10–12). In the UK and elsewhere, declines in wild spat availability have raised concerns over the long-term sustainability of mussel farming (9). These changes, along with increasing ocean temperatures, coincide with poleward range shifts (13), potentially leading to weakened immune responses, and greater susceptibility to disease (14, 15). Disseminated neoplasia and pathogenic *Vibrio* species continue to pose persistent challenges, particularly in hybrid zones of the *Mytilus* complex where immunological consequences of introgression remain unclear (16–19). Taken together, these findings underscore the importance of better understanding the effects of environmental stressors on the immune functioning of *Mytilus* spp.

Mussels rely on mechanosensory detection of waterborne vibrations, and exhibit a range of physiological responses to acoustic stress. These include reduced filtration rates, increased oxidative stress, and impaired immune function (1, 3). Previous studies on *Mytilus edulis* indicate that noise exposure can induce DNA damage, alter haemocyte function, and suppress metabolic activity (1, 2). Hubert et al. (3) demonstrated that acoustic disturbance leads to valve closure and potential disruption of feeding behaviour, which may exacerbate physiological stress. Similarly, a study by Vazzana et al. (20) showed an increase in the number of *Mytilus* haemocytes following noise exposure, however their cytotoxicity was reduced with decreased peroxidase activity, pointing to a decoupling between immune cell recruitment and function.

Given the crucial role of immune responses in host defence against pathogenic infections, understanding how noise exposure influences immune responses is essential. In this study, we investigated the effects of noise exposure on immune responses in *Mytilus* spp. by combining transcriptional profiling with a functional bacterial challenge. Mussels were exposed to underwater ship noise played on a 12-hour cycle for 7 days, allowed a day to recover, then subjected to a bath challenge with a GFP-tagged strain of *V.* sp*lendidus*. Samples were collected at multiple time points during and after noise exposure, as well as following bacterial challenge, to assess transcriptional dynamics and bacterial burden in the haemolymph.

Together, these findings highlight an underappreciated dimension of anthropogenic noise as an immune-modulatory factor in marine invertebrates, with potential relevance for both ecological dynamics and aquaculture practices.

## Materials and methods

### Animals

Adult mussels (*M. edulis* and *M. galloprovincialis*, hybrids hereafter referred to as *Mytilus* spp. (21)), of approximately 7 cm shell-length, were obtained from a commercial mussel farm (Shetland, UK) via the Scottish Shellfish Marketing Group (SSMG, Bellshill, UK). Mussels were allowed acclimation of at least one week prior to experiments (n=446). They were maintained at the Roslin Institute in a flow through artificial seawater (ASW) system with constant aeration maintained at 15°C with a salinity of 30–35 ppt and a pH of 7.5-8.5. The mussels were fed daily during acclimation and noise exposure with 1ml of liquid algae concentrate, PhytoBloom^®^ Shellbreed (Necton, Portugal), containing *Skeletonema* sp.*, Tisochrysis lutea (T-iso)*, and *Tetraselmis* sp. The system was maintained under the same conditions throughout the experiment, but no feed was provided during bacterial challenge.

### Experimental design

**Figure 1:**
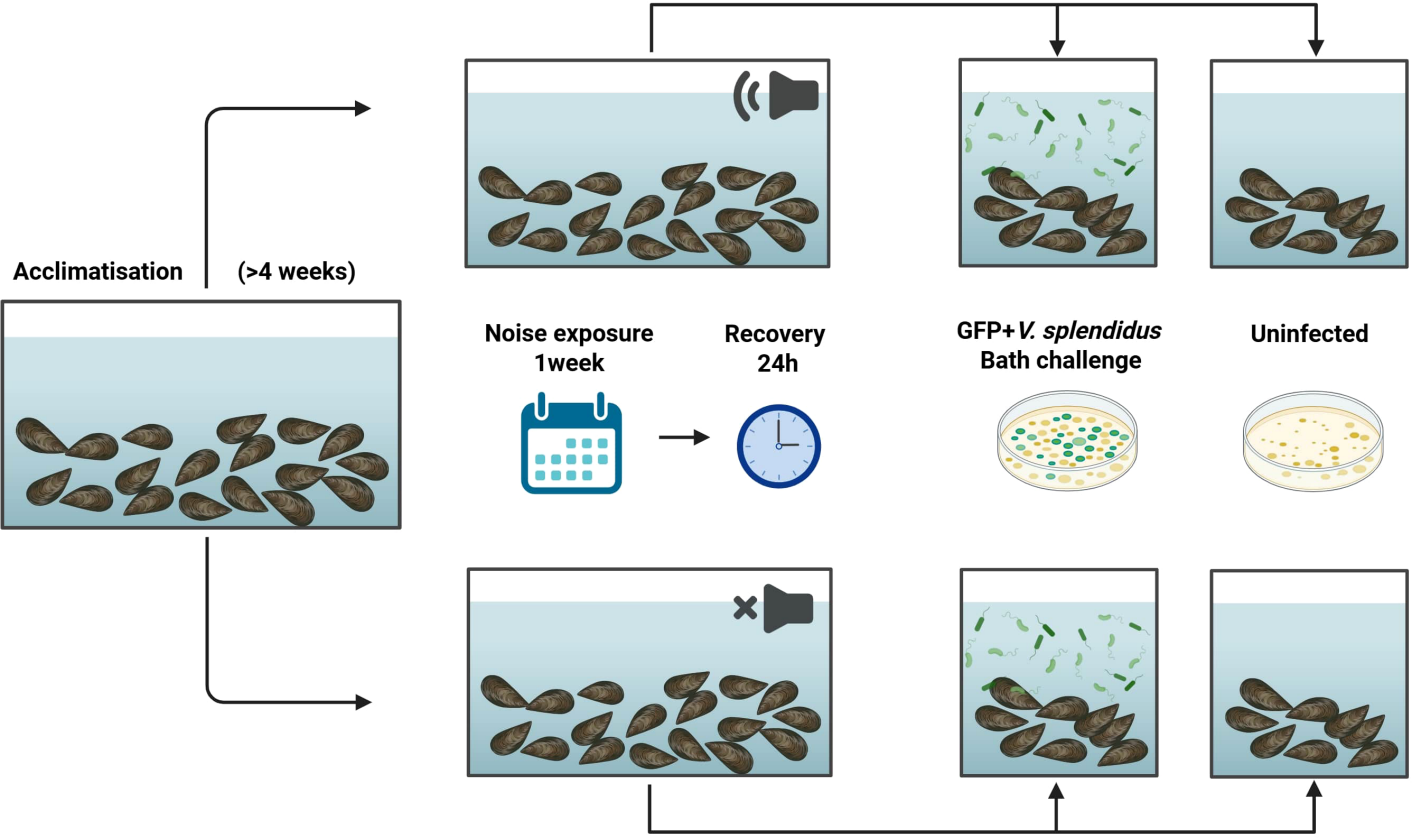
Schematic of the experiment Schematic of the experimental design. Mussels were exposed to ship noise for 7 days in a 12 h on/off cycle. On day 7, all groups were subjected to a bath challenge with GFP-labelled *Vibrio* sp*lendidus* or mock infection. Transcriptomic and bacterial burden measurements were collected at multiple time points. **Figure 1** was created with BioRender.com.

A tank of mussels were exposed to underwater ship noise for seven days (12-hour noise cycles, n=208), while another tank for the control mussels had a speaker but no noise was played (n=208). Both groups (noise-exposed and non-noise) were then separated into infected and uninfected controls (n=101 in each tank). The infection was carried out via a 24-hour bath challenge with 2x10^7^ CFU/ml GFP+ *V.* sp*lendidus.* This concentration was chosen to assess host immune responses without inducing overt disease, and is within the range used in comparable studies e.g., Saco et al., 2020, used 1×10^8^ CFU/ml to induce mortality (22). Mussels were held in a flow-through system during noise exposure and transferred to static water for the bacterial challenge (**Figure 1**).

### Noise exposure

**Figure 2:**
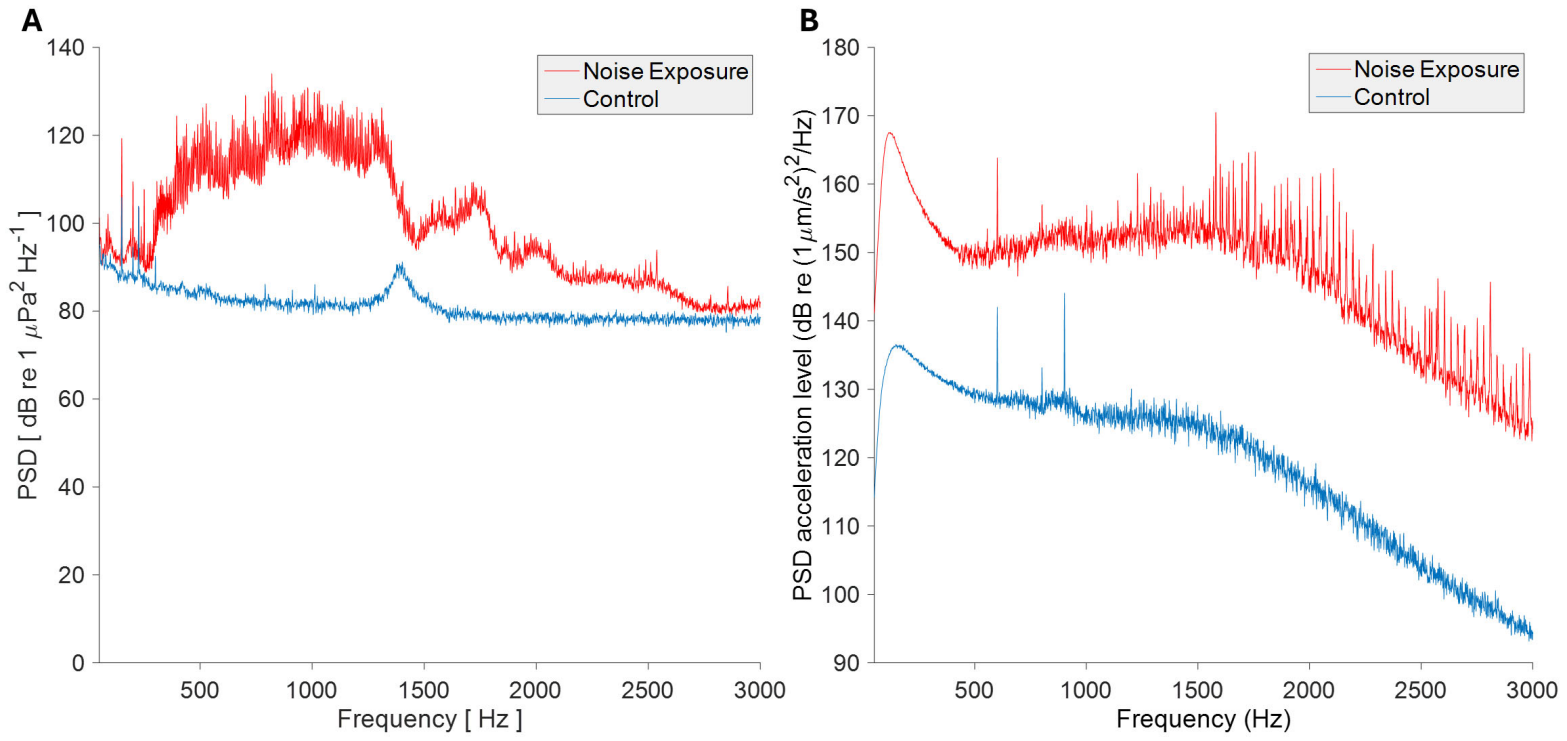
Analysis of acoustic stimuli and sound playback conditions. Mean power spectral density of 30 s of each sound condition of **(A)** acoustic pressure and **(B)** particle acceleration, for control and exposure conditions. Analysis performed in MATLAB R2015b (pressure) and MATLAB Compiler Runtime R2013a (particle acceleration). fft lengths = 48 kHz (pressure) and 44.1 kHz (particle acceleration), both resulting in 1 Hz bands.

Noise exposure consisted of ship passage recordings from three UK ports, produced previously (23), randomized over a 12-hour exposure period. These playback sounds were calibrated to reflect the noise levels encountered at a distance of roughly 200–300 meters from a vessel, based on previous studies (24, 25), and were played for durations consistent with typical exposure times in frequently trafficked shipping routes. These tracks were played back as WAV files via a Laptop; amplifier (Pioneer A-10-K, 50W, frequency response: 20-20,000 Hz, Pioneer Corporation, Tokyo, Japan); and Clark Synthesis AQ339 underwater speaker (effective frequency range 20-17,000 Hz, Clark Synthesis Inc., Littleton, CO, U.S.A) in a controlled tank environment. Control tanks experienced identical conditions without noise playback. Sound levels were recorded at the mussels’ position in the experimental tanks using a HiTech HTI-94-SSQ hydrophone with inbuilt preamplifier (High Tech, Inc., USA) for pressure or a custom calibrated sensor (suspended triaxial accelerometer potted in epoxy resin, as described previously (1), for particle acceleration, attached to a Zoom H6 Portable Recorder (Zoom Corp, Tokyo, UK). Sound pressure peaked at 134 dB (re 1 µPa^2^ Hz^-1^) for the noise exposure and 106 dB (re 1 µPa^2^ Hz^-1^) for control conditions, as measured in PAMGuide (Merchant et al., 2015) (**Figure 2A**). Particle acceleration, measured in paPAM (Nedelec et al., 2016), peaked at 170 dB (re 1 (µm/s^2^)^2^ Hz) for noise exposure and 144 dB (re 1 (µm/s^2^)^2^ Hz) for control conditions (**Figure 2B**).

### Construction of GFP-tagged *V. splendidus*

GFP-tagged *Vibrio* sp*lendidus* strain C04a (26) was generated using a triparental mating approach following the protocol of Travers et al. (27) and Stabb and Ruby (28). Briefly, donor and helper *E. coli* strains harbouring plasmids pVSV102 (GFP, kanamycin resistance) and pVSV104 (helper plasmid) were grown overnight in LB with kanamycin. *E. coli* helper (CC118 λpir) and donor (DH5a) strains containing these plasmids were received as a gift from the lab of Prof. Eric Stabb at the University of Illinois Chicago. Stationary-phase cultures were mixed with recipient *V.* sp*lendidus* and spotted onto LBS agar for conjugation at 28°C. After overnight incubation, cells were resuspended, diluted, and plated onto selective marine agar. Transconjugants were selected based on kanamycin resistance and confirmed by GFP fluorescence and PCR screening.

### Bacterial challenge and quantification

GFP-labelled *V.* sp*lendidus* (C04a) cultures were grown to an OD of 1.0 overnight, shaking at 180 rpm in marine broth (Merck) at 22°C. Prior to infection, the culture was washed twice in ASW. Infections were performed using 2x10^7^ cfu/ml in 10 L of ASW in static immersion tanks (n=101). A mock infection was carried out on control mussels with no bacteria added to their tanks (n=101). The concentration of bacteria used was confirmed by performing serial dilutions and colony counts on marine agar. The timepoint of 24 hours after exposure was used to study the immune response as this time point has previously been shown to generate a robust response to *V.* sp*lendidus* in *Mytilus* spp. (22, 29). Bacterial burden was quantified at 24 h post infection via haemolymph extraction, serial dilution in sterile artificial seawater, and plating on marine agar (Merck) in triplicate. GFP+ colonies were counted under blue light to determine infection prevalence. Statistical analysis was conducted using Student’s t-test. Gill tissue from infected and uninfected mussels in control and noise-exposed groups was also sampled from each tank 24 hours following infection (n=6 individuals sampled for each group).

### RNA sequencing and transcriptomic analysis

Gill tissue was dissected from mussels following 4 hours, 24 hours and 7 days of noise exposure and again at 48 hours recovery after the noise was turned off (n=3 individuals sampled for each time point). Mussels were also sampled at 24 hours post-*Vibrio* challenge (n=6 individuals sampled for both infected and uninfected). RNA was extracted from gill tissue using the RNeasy Blood & Tissue Kit (Qiagen) according to manufacturer’s instructions. Strand-specific RNA sequencing was performed on the NovaSeq XPLUS platform (Illumina PE150) at 30M reads per sample. Raw reads were deposited in the EMBL-EBI Array Express database with accession numbers E-MTAB-15321 and E-MTAB-15323. Fastp (v0.24.0) was used to trim adapters and filter for PHRED score >15 and length >30. The annotated transcriptome from the xbMytEdul2.2 assembly (GCF_963676685.1) was used to map reads using default parameters for stranded mapping with Kallisto (v0.44.0). Differentially expressed genes were identified using DESeq2. All code used is available on Github repository https://github.com/Roslin-Aquaculture/Mussel_Noise_RNAseq/. DEGs with P < 0.05 were compared between treatments groups using Venny 2.1 (30). Full lists of these genes are available in the **Supplementary Table**.

## Results

We first examined whether noise exposure influenced mussel survival either alone or in combination with *Vibrio* sp*lendidus* challenge. During the 7-day noise exposure period, no mortality was observed in either control or noise-exposed groups (**Supplementary Figure 1a**). Following bacterial bath challenge on day 7, survival was tracked over the subsequent 7 days in four treatment groups: control (no noise, no *Vibrio*), noise-only, *Vibrio*-only, and noise + *Vibrio*. No significant difference in cumulative mortality was detected between groups (**Supplementary Figure 1b**, log-rank test, p > 0.05), indicating that noise exposure did not exacerbate mortality, even following bacterial infection.

**Figure 3:**
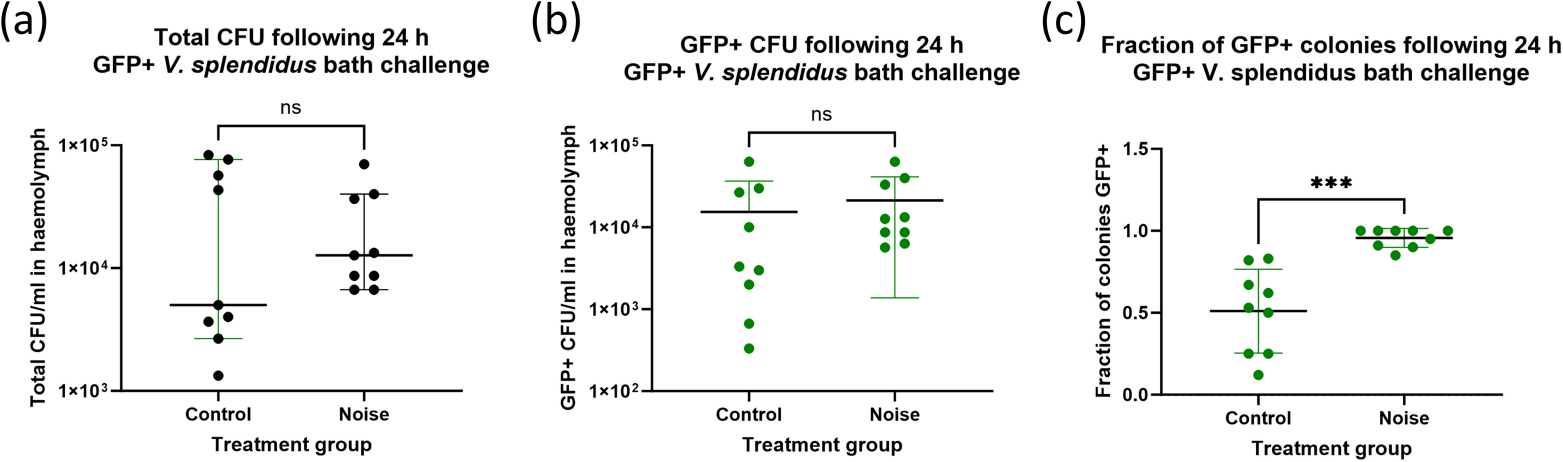
Bacterial burden in mussel haemolymph 24 h after *Vibrio* sp*lendidus* challenge. Quantification of bacterial burden in mussel haemolymph following serial dilution and growth on marine agar. Shown are **(a)** Total colony-forming units (CFU/ml) recovered from mussel haemolymph, **(b)** GFP-positive *V.* sp*lendidus* CFU/ml only and **(c)** the overall fraction of GFP-positive colonies. Each point represents an individual mussel (n = 6 per group). Bars show mean ± SD.

To assess whether noise exposure altered the ability of mussels to control bacterial infection, haemolymph samples were plated on marine agar 24 hours after bacterial challenge. Neither the total bacterial burden, nor the number of GFP-positive *V.* sp*lendidus* colonies (CFU/ml) was significantly different between control and noise-exposed mussels (**Figures 3a, b**). However, the fraction of GFP+ colonies relative to total CFU (**Figure 3c**) was significantly different between control and noise-exposed groups, suggesting that while bacterial infection occurred in both groups, noise exposure may lead to higher rates of infection.

**Figure 4:**
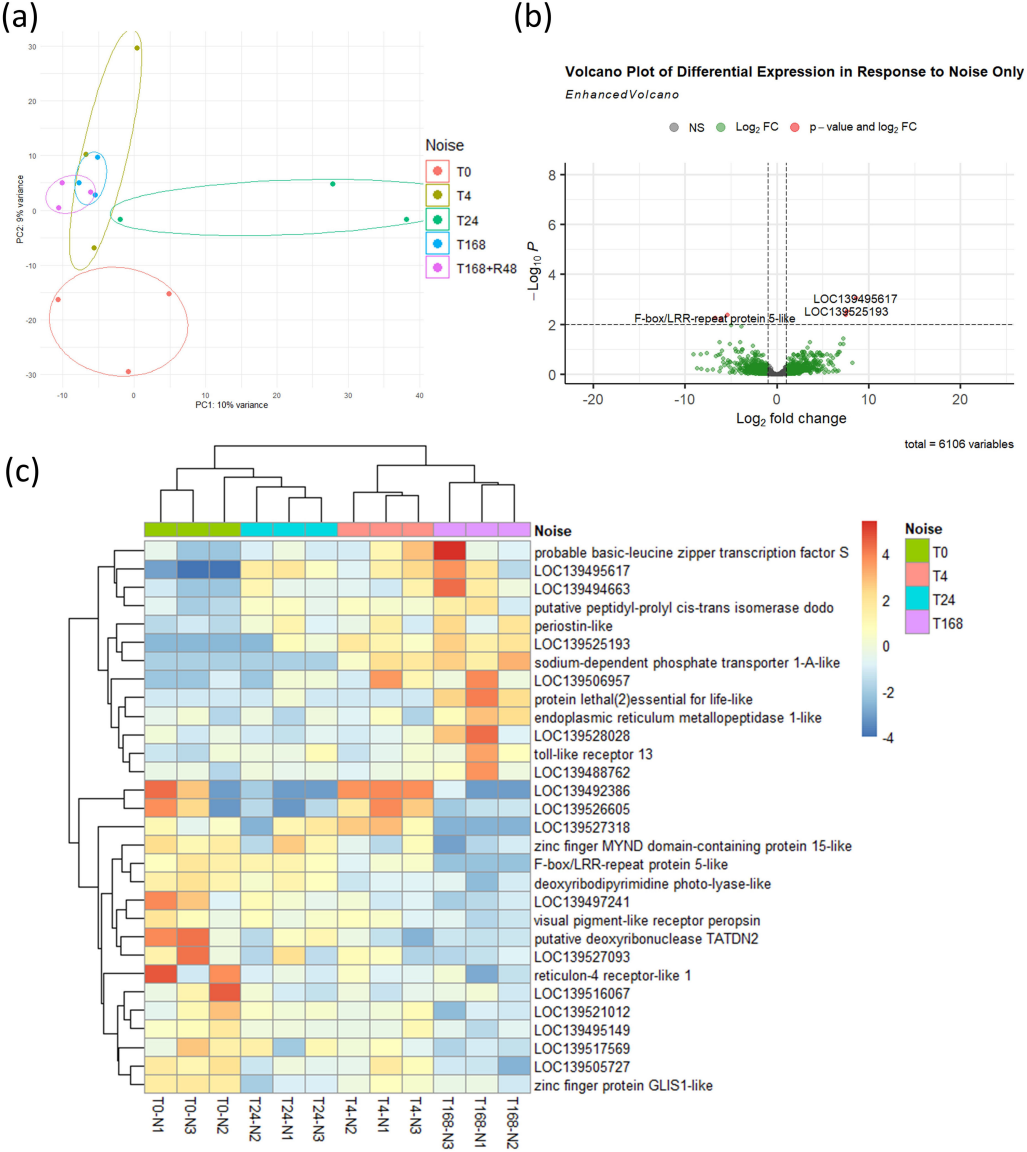
Transcriptomic response to noise exposure. **(a)** PCA of mussel transcriptomes at baseline (T0), 4 h (T4), 24 h (T24), 7 days (T168), and 48 h recovery post-noise (R48). n = 3 per time point. Ellipses were manually drawn using group range along PC axes. **(b)** Volcano plot showing 6 DEGs (threshold: *p* < 0.01) between noise-exposed groups (T4, T24, T168) and baseline (T0). **(c)** Heatmap of 30 most significant DEGs across time points.

We next explored the transcriptional response of *Mytilus* spp. to ship noise using RNA sequencing at multiple time points: prior to exposure (T0), 4 hours (T4), 24 hours (T24), and 7 days (T168) post-noise onset. A recovery group was also sampled after 48 hours of silence following 7 days of exposure (termed R48). PCA revealed a subtle but progressive shift in gene expression with noise duration, which largely returned toward baseline in the recovery group (**Figure 4a**). Differential expression analysis across all time points revealed a small number of genes (*p* < 0.01) differentially expressed in noise-exposed mussels relative to T0 controls (**Figure 4b**). A heatmap of the 30 most significantly altered transcripts shows relatively consistent patterns across exposure time points (**Figure 4c**), indicating that the noise-induced transcriptional response is mild.

**Figure 5:**
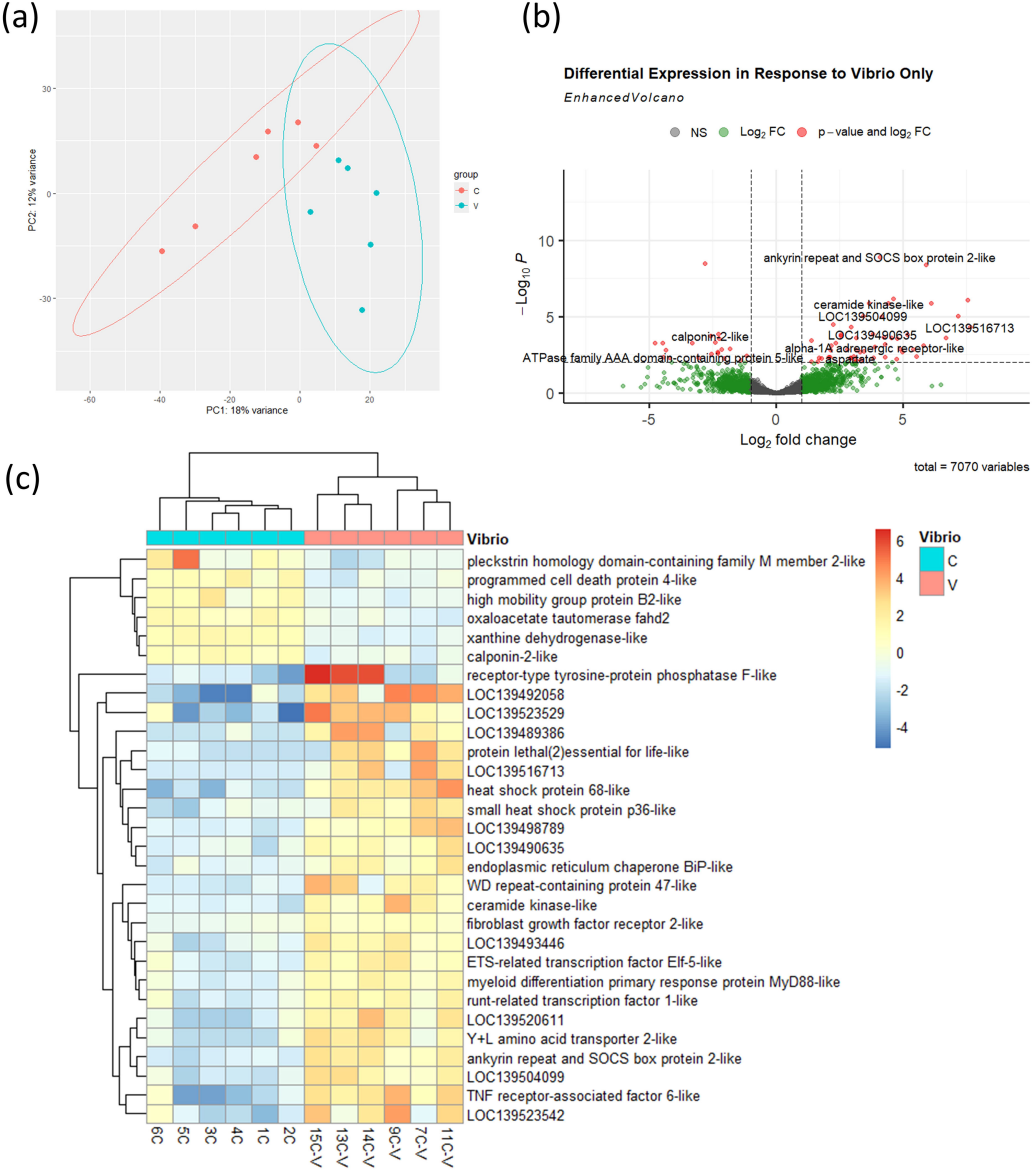
Transcriptional response to *V.* sp*lendidus* infection in control mussels. **(a)** PCA of transcriptomes from control (C) and infected (V) mussels, n = 6 per group. Ellipses were generated using stat_ellipse() based on a multivariate normal distribution. **(b)** Volcano plot showing 72 DEGs (threshold: *p* < 0.01) between infected and uninfected control mussels. **(c)** Heatmap showing expression profiles of 30 most significant DEGs.

To establish a baseline immune response to *V.* sp*lendidus*, we performed transcriptomic analysis in control mussels (not exposed to noise) 24 hours after bacterial challenge. PCA revealed a clear separation between infected (V) and uninfected (C) mussels (**Figure 5a**), with hundreds of differentially expressed genes (DEGs) identified (**Figure 5b**). The expression profile included upregulation of known immune-related genes, including pattern recognition receptors, signalling molecules, and antimicrobial effectors. Heatmap clustering of the top 30 DEGs further confirmed the distinct immune signature induced by *V.* sp*lendidus* infection in mussels which were not exposed to noise (**Figure 5c**).

**Figure 6:**
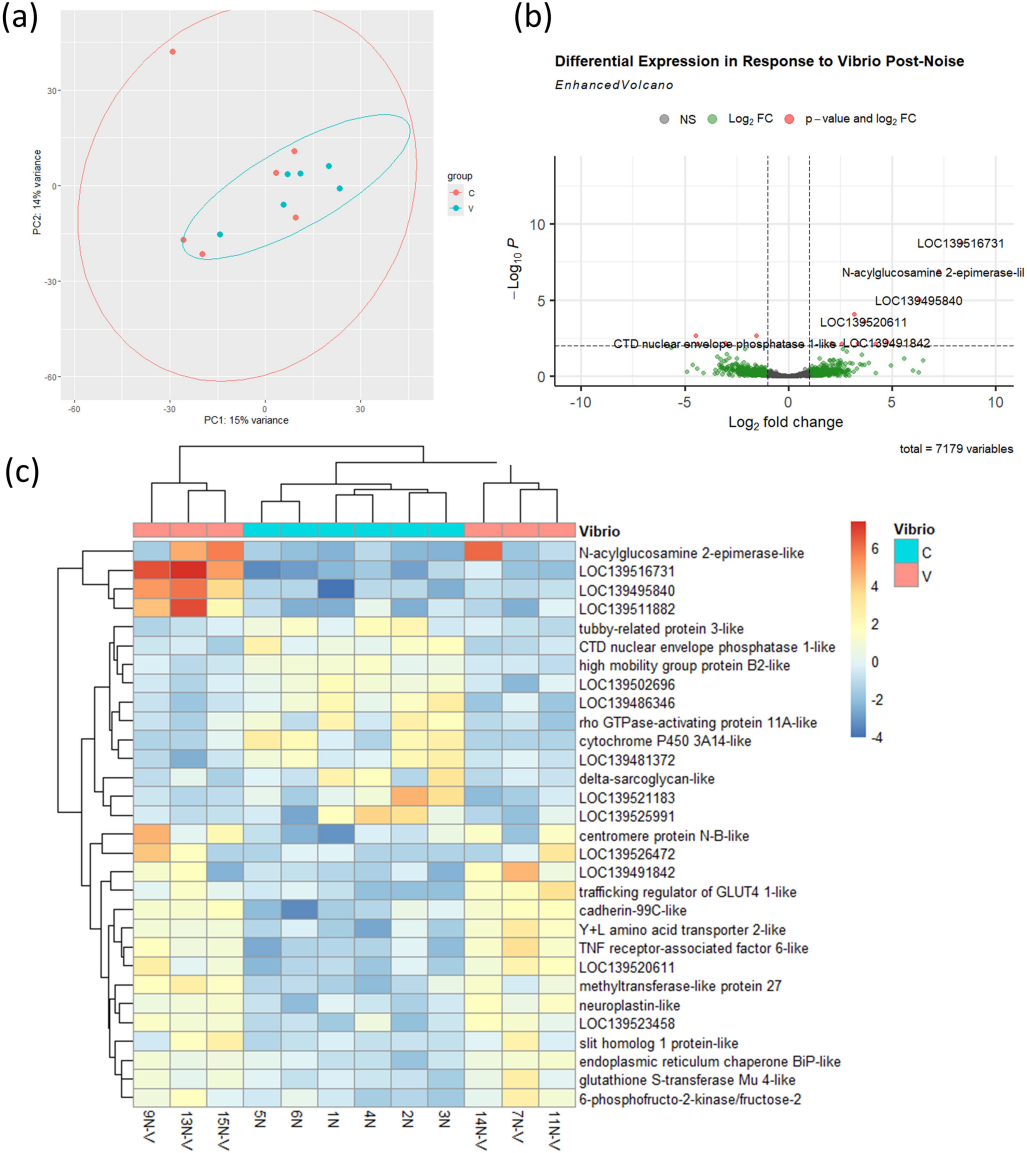
Transcriptional response to *V.* sp*lendidus* infection in noise-exposed mussels. **(a)** PCA of transcriptomes from noise-exposed uninfected **(C)** and infected (V) mussels, n = 6 per group. Ellipses were generated using stat_ellipse() based on a multivariate normal distribution. **(b)** Volcano plot of 14 DEGs (threshold: *p* < 0.01) between infected and uninfected mussels. **(c)** Heatmap of 30 most significant DEGs.

We then examined whether prior exposure to ship noise altered the immune transcriptional response to *V.* sp*lendidus*. Contrasting with results in **Figure 5**, PCA (**Figure 6a**) showed virtually no separation between infected and uninfected samples in the noise-exposed cohort. Differential expression analysis revealed much fewer DEGs in noise-exposed mussels (**Figure 6b**), with reduced induction of typical immune markers. Heatmap clustering further confirmed the attenuated nature of the transcriptional response following V. splendidus infection in noise-exposed mussels (**Figure 6c**), with less clear grouping of the uninfected and infected groups.

**Figure 7:**
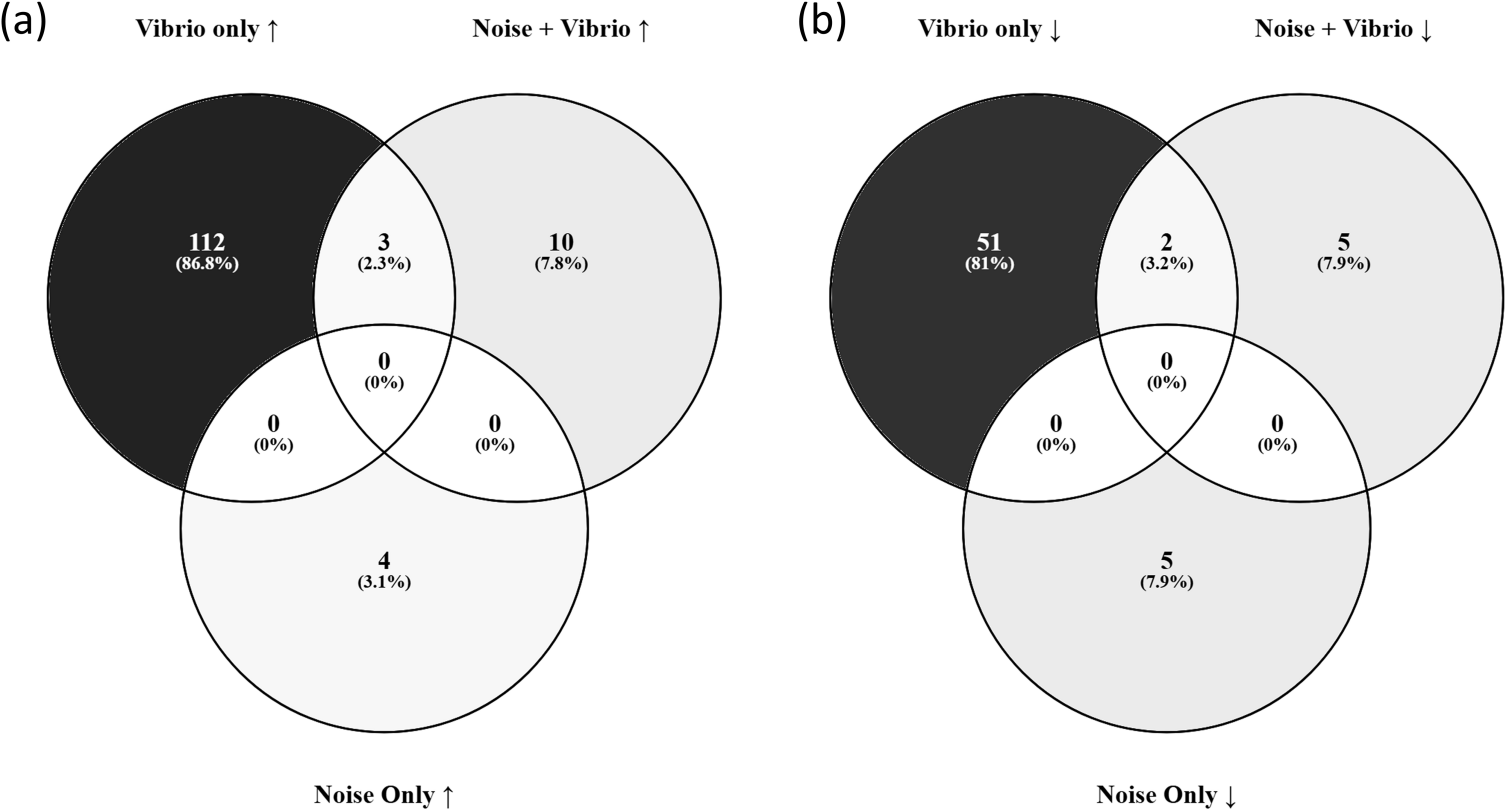
Venn diagrams comparing DEGs in response to *V.* sp*lendidus* and/or noise exposure. **(a)** Upregulated genes in response to “Vibrio only”, “Noise + Vibrio”, and “Noise Only” contrasts, *p*<0.05. **(b)** Downregulated genes (*p*<0.05) in the same contrasts.

To compare the transcriptomic responses across treatments, we generated Venn diagrams of up- and downregulated DEGs in three contrasts: (i) *V.* sp*lendidus* infection in control mussels which had not been exposed to noise (Vibrio only), (ii) *V.* sp*lendidus* infection in noise-exposed mussels (Noise + Vibrio), and (iii) noise exposure alone (Noise Only) with a threshold of *p*<0.5. This resulted in a total number of 168 DEGs for response to “Vibrio only” compared with 20 in response to “Noise + Vibrio” and 9 in response to “Noise Only” (**Figure 7**). The overlap of upregulated genes (**Figure 7a**) was limited, with a distinct set of genes induced only in the “Vibrio only” group, many of which were not activated following infection in noise-exposed mussels. A similar pattern was observed for downregulated genes (**Figure 7b**), indicating that noise alters both the magnitude and composition of the mussel immune transcriptional response.

**Figure 8:**
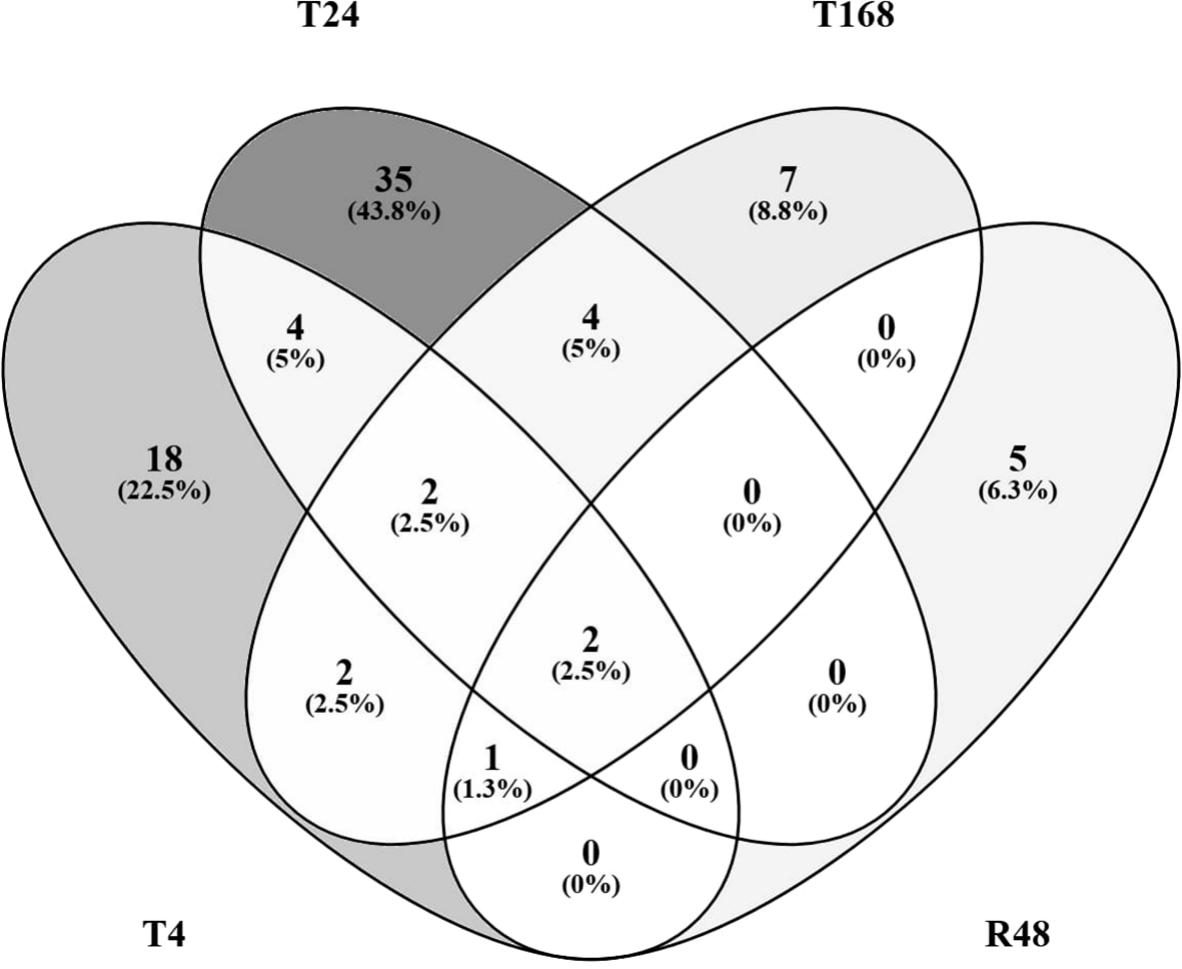
Venn diagrams comparing DEGs in noise exposure only. Differentially Expressed Genes (*p*<0.05, |ln|>1), either up- or downregulated in response to noise only compared to control at T0 at either 4 h (T4), 24 h, or 168 h of noise exposure, or 48 h of recovery following 168 h of noise exposure (R48).

We also generated Venn diagrams comparing the DEGs in response to noise only at each of the 3 time points during noise exposure (T4, T24 and T168) and the 48 h recovery period after 7 days of noise exposure (R48) compared with the control (**Figure 8**).

## Discussion

At 24 hours post-*Vibrio* challenge, mussels that had not been previously exposed to noise exhibited a robust transcriptional activation of immune pathways, consistent with a typical response to pathogenic stimulation displaying 168 DEGs overall. In contrast, mussels that experienced prior noise exposure demonstrated a markedly attenuated immune response, with only 20 DEGs in total, suggesting that the acoustic stress may impair host capacity to mount an effective defence.

The principal component analysis of samples collected at multiple time points during and following noise exposure revealed a shift in gene expression patterns driven by time. A deviation from baseline was detectable as early as 4 hours post-exposure, with maximal divergence observed at 24 hours. Several genes implicated in immune and stress responses displayed differential regulation at this 24-hour time point (see **Supplementary Table**). Among upregulated transcripts were GRB2-associated and regulator of MAPK protein-like, suggesting potential activation of MAPK signalling pathways relevant to immune modulation (31). Cytochrome P450 12a5 and oxysterol-binding protein 1-like were also elevated, indicating possible shifts in xenobiotic metabolism and cellular lipid signalling under stress (32, 33). Conversely, downregulated genes included phosphatidylinositol 3-kinase catalytic subunit type 3-like (PI3K), which plays a role in immune cell signalling and survival (34). Additional stress-linked downregulated genes included acidic phospholipase A2, dynein assembly factor WD repeat-containing 1-like, reticulon-4 receptor-like 1, signal peptide peptidase-like 2A, and Nek7-like kinase – many of which are involved in vesicular transport, ER stress response, and cell cycle regulation (22, 35–37).

Notably, mussels sampled after seven days of continuous noise exposure (T168), as well as those given 48 hours of recovery following noise cessation (R48), exhibited transcriptional profiles closer to those of the control group. However, despite this apparent return to baseline at the transcriptomic level, the functional immune response to bacterial challenge remained altered. This could indicate that regulation of immune competence is driven through mechanisms such as epigenetics, or in part by shifts in metabolic activity.

It was also noted that when analysing the noise-exposed mussels as a single group across time points (**Figures 4B, C**), TLR13 was the only immune-related gene identified among the most significant DEGs. This suggests that immune modulation may be subtle or timepoint-specific rather than a dominant feature of the overall response to noise alone.

The *V.* sp*lendidus* strain used in this study was originally isolated from a mortality event in Pacific oysters, *Crassostrea gigas*, in Scotland (26). While this strain is not known to be directly pathogenic to *Mytilus* spp., it represents an environmentally relevant opportunistic bacteria and was selected to assess host immune competence rather than to induce disease. Opportunistic pathogens such as *V.* sp*lendidus* are typically controlled by a functional immune system, thus immune impairment may be revealed by a failure to mount an appropriate response to such exposures (38). The relatively modest transcriptional response we observed following *Vibrio* challenge, compared with previous studies using more virulent strains (22), likely reflects both the lower pathogenicity of our isolate and the lower bacterial dose used in this experiment (10^7^ CFU/ml vs. 10^8^ CFU/ml). Nonetheless, clear differences between noise-exposed and control mussels highlight the value of using sublethal bacterial challenges to detect functional immune changes.

Although no significant difference was observed in total bacterial burden between groups, all colonies recovered from the haemolymph of noise-exposed mussels were GFP-positive *Vibrio*, whereas approximately half of the colonies from the control group were not. This may either suggest a selective impairment in preventing early infection following acoustic stress, or potentially an increase in filtration by the noise-exposed mussels, reflective of metabolic stress.

While enrichment of GO terms was limited or inconsistent across most comparisons, notable DEGs were up- or down-regulated in response to *Vibrio* exposure, both with and without prior noise stress. Several immune-related genes upregulated following *Vibrio* challenge alone included MyD88-like proteins that are a key adaptor molecule in the Toll-like receptor (TLR) signalling pathways. MyD88 has also been studied in molluscs revealing unique evolutionary patterns and functional roles in immune signalling (39, 40). NF-κB p105 subunit-like, another protein that plays a central role in immune signalling and defence in vertebrates and marine invertebrates (41). Other upregulated genes included peptidoglycan recognition protein 1 (PGRP1)-like a key component of the innate immune system in molluscs, potentially acting as a pattern recognition receptor that detects bacterial cell wall components. Studies have identified and characterized several PGRP1-like proteins in different mollusc species, highlighting their structural features, expression patterns, and roles in antibacterial defence (42–44). Similarly, Toll-like receptor 13 (TLR13) and tumour necrosis-associated factor 6 (TRAF6) (45) are key components of the innate immune system. TLR13 acts as a pattern recognition receptor, detecting pathogen-associated molecular patterns and initiating immune responses. These include activation of MyD88, TRAF6 and NF-κB (46–48). Also upregulated was TNFAIP3-interacting protein 2-like (TNIP2), a protein involved in regulating inflammation, cell death, and RNA metabolism (49, 50). Several stress-responsive genes were also differentially expressed, including HSP68-like, HSP70 B2-like, and small HSP p36-like. HSP68-like is known for its role in cellular protection, particularly under changing salinity conditions, and is associated with endoplasmic reticulum chaperone BiP-like (GRP78) (51). These changes are consistent with cellular stress and protein folding demands associated with infection (50, 52–54). PARP1, a DNA repair enzyme also involved in inflammatory responses, was downregulated in response to *Vibrio* (55, 56). In contrast, the transcriptomic response to *Vibrio* in noise-exposed mussels was minimal, with TRAF6 as the only immune-related gene showing significant upregulation. HMGB2, a chromatin-associated protein linked to DNA repair and immune activation, was downregulated, further suggesting altered immune readiness and resistance to bacterial infection (57).

These findings are consistent with growing evidence that anthropogenic noise can act as a physiological stressor in marine invertebrates (1–3). Previous studies have reported reduced haemocyte activity, elevated oxidative stress markers, and altered behaviour (e.g., changes in valve closure) in noise-exposed mussels (1). Increased valve closure rates in noise-exposed mussels, as reported by Hubert et al. (3), may contribute to altered immune responses by limiting exposure to environmental microbial signals. Researchers working on *M. galloprovincialis* found that noise exposure led to an increased number of haemocytes, but these displayed reduced cytotoxic activity with lower levels of peroxidase (20, 58). It should be noted that the reference genome used in our analysis was derived from a *Mytilus edulis* individual. While this fully annotated reference is the most genetically appropriate assembly currently available for our study population, high levels of gene presence–absence variation (PAV) have been documented across *Mytilus* spp. (59–62). As such, some stress- or immune-related genes may have been absent from either the reference or the study individuals, potentially influencing downstream interpretation. Nevertheless, the transcriptomic data presented here provide a molecular framework for these observations and suggest that noise exposure may alter immune gene regulation, potentially through stress hormone signalling, redox imbalance, or neuroendocrine modulation – mechanisms that warrant further investigation.

From an applied perspective, the persistence of immune alteration despite transcriptional return to baseline raises relevant questions for shellfish aquaculture. With increasing vessel traffic and offshore development, mussels and other aquatic species may be subject to chronic or intermittent acoustic disturbances. While our data suggest that noise exposure can modulate immune responses, the long-term implications for disease resistance and productivity remain unclear. Importantly, the transient transcriptomic effects and persistent immune alteration we observed in response to noise is consistent with patterns seen under other environmental stressors such as thermal or oxidative stress. This suggests that noise functions as a physiological stressor, eliciting classical features of stress-induced immune modulation. It is possible that with extended exposure or acclimation, mussels might regain full immunocompetence, but this has yet to be demonstrated. Indeed, on offshore wind structures, mussel biomass can exceed 3.4 kg m^-2^ (63), transforming artificial substrates into rich secondary habitats. Future work should therefore assess both the duration and reversibility of noise-induced immune modulation, to better inform aquaculture management strategies, including site selection and noise mitigation.

This study demonstrates that exposure to anthropogenic noise suppresses the transcriptional immune response of *Mytilus* spp. to bacterial challenge. While gene expression profiles appeared to normalize following extended exposure or recovery, immune competence remained impaired, indicating a decoupling of transcriptomic recovery and functional immunity. These findings highlight the immunomodulatory potential of acoustic stress in marine invertebrates and underscore the need for further mechanistic studies to determine the pathways involved. In the context of sustainable aquaculture, our results suggest that environmental noise may be an underappreciated factor influencing host–pathogen dynamics and mussel health in farmed settings.

## Data Availability

The datasets presented in this study can be found in online repositories. The names of the repository/repositories and accession number(s) can be found below: https://www.ebi.ac.uk/arrayexpress/, E-MTAB-15321 https://www.ebi.ac.uk/arrayexpress/, E-MTAB-15323.

## Supplementary material

Supplementary Figure 1 Mussel survival during noise exposure and after Vibrio challenge. **(a)** Survival during the 7-day noise exposure period(n = 208 mussels per tank, 1 tank per group). **(b)** Survival during the week following *Vibrio* or mock challenge, across four groups: Control, Noise Only, *Vibrio* Only, and Noise + *Vibrio* (n = 101 mussels per tank, 1 tank per group). Dotted lines represent 95% confidence intervals for survival estimates, calculated by GraphPad Prism using the Kaplan-Meier method. No significant differences in mortality were observed (log-rank test, p > 0.05).

## References

[1] Wale MA Briers RA Hartl MGJ Bryson D Diele K. From DNA to ecological performance: Effects of anthropogenic noise on a reef-building mussel. Sci Total Environ. (2019) 689:126–32. doi: 10.1016/j.scitotenv.2019.06.380, PMID: 31271981

[2] Zhao X Sun S Shi W Sun X Zhang Y Zhu L. Mussel byssal attachment weakened by anthropogenic noise. Front Mar Sci. (2021) 8:821019. doi: 10.3389/fmars.2021.821019

[3] Hubert J Moens R Witbaard R Slabbekoorn H. Acoustic disturbance in blue mussels: sound-induced valve closure varies with pulse train speed but does not affect phytoplankton clearance rate. ICES J Mar Sci. (2022) 79:2540–51. doi: 10.1093/icesjms/fsac193

[4] Norling P Kautsky N. Structural and functional effects of Mytilus edulis on diversity of associated species and ecosystem functioning. Mar Ecol Prog Ser. (2007) 351:163–75. doi: 10.3354/meps07033

[5] Chase M Jones SH Hennigar P Sowles J Harding G Freeman K. Gulfwatch: Monitoring spatial and temporal patterns of trace metal and organic contaminants in the Gulf of Maine (1991–1997) with the blue mussel, Mytilus edulis L. Mar Pollut Bull. (2001) 42:490–504. doi: 10.1016/S0025-326X(00)00193-4, PMID: 11468927

[6] MacDonald BA Robinson SM Barrington KA. Feeding activity of mussels (Mytilus edulis) held in the field at an integrated multi-trophic aquaculture (IMTA) site (Salmo salar) and exposed to fish food in the laboratory. Aquaculture. (2011) 314:244–51. doi: 10.1016/j.aquaculture.2011.01.045

[7] Broszeit S Hattam C Beaumont N. Bioremediation of waste under ocean acidification: reviewing the role of Mytilus edulis. Mar pollut Bull. (2016) 103:5–14. doi: 10.1016/j.marpolbul.2015.12.040, PMID: 26778338

[8] Pauly D Zeller D. Comments on FAOs state of world fisheries and aquaculture (SOFIA 2016). Mar Policy. (2017) 77:176–81. doi: 10.1016/j.marpol.2017.01.006

[9] Regan T Bean TP Ellis T Davie A Carboni S Migaud H. Genetic improvement technologies to support the sustainable growth of UK aquaculture. Rev Aquacult. (2021) 13:1958–85. doi: 10.1111/raq.12553

[10] Jones SJ Lima FP Wethey DS. Rising environmental temperatures and biogeography: poleward range contraction of the blue mussel, Mytilus edulis L., in the western Atlantic. J Biogeog. (2010) 37:2243–59. doi: 10.1111/j.1365-2699.2010.02386.x

[11] Ellis RP Widdicombe S Parry H Hutchinson TH Spicer JI. Pathogenic challenge reveals immune trade-off in mussels exposed to reduced seawater pH and increased temperature. J Exp Mar Biol Ecol. (2015) 462:83–9. doi: 10.1016/j.jembe.2014.10.015

[12] Cano I Parker A Ward GM Green M Ross S Bignell J. First detection of Francisella halioticida infecting a wild population of blue mussels Mytilus edulis in the United Kingdom. Pathogens. (2022) 11:329. doi: 10.3390/pathogens11030329, PMID: 35335653 PMC8953295

[13] Seuront L Nicastro KR Zardi GI Goberville E. Decreased thermal tolerance under recurrent heat stress conditions explains summer mass mortality of the blue mussel Mytilus edulis. Sci Rep. (2019) 9:17498. doi: 10.1038/s41598-019-53580-w, PMID: 31767954 PMC6877631

[14] Ripabelli G Sammarco ML Grasso GM Fanelli I Caprioli A Luzzi I. Occurrence of Vibrio and other pathogenic bacteria in Mytilus galloprovincialis (mussels) harvested from Adriatic Sea, Italy. Int J Food Microbiol. (1999) 49:43–8. doi: 10.1016/S0168-1605(99)00056-2, PMID: 10477069

[15] Eggermont M Bossier P Pande GSJ Delahaut V Rayhan AM Gupta N. Isolation of Vibrionaceae from wild blue mussel (Mytilus edulis) adults and their impact on blue mussel larviculture. FEMS Microbiol Ecol. (2017) 93:fix039. doi: 10.1093/femsec/fix039, PMID: 28334251

[16] Fraïsse C Roux C Welch JJ Bierne N. Gene-flow in a mosaic hybrid zone: is local introgression adaptive? Genetics. (2014) 197:939–51. doi: 10.1534/genetics.114.161380, PMID: 24788603 PMC4096372

[17] Burioli EA Hammel M Bierne N Thomas F Houssin M Destoumieux-Garzón D. Traits of a mussel transmissible cancer are reminiscent of a parasitic life style. Sci Rep. (2021) 11:24110. doi: 10.1038/s41598-021-03598-w, PMID: 34916573 PMC8677744

[18] Michalek K Vendrami DL Bekaert M Green DH Last KS Telesca L. Mytilus trossulus introgression and consequences for shell traits in longline cultivated mussels. Evol Appl. (2021) 14:1830–43. doi: 10.1111/eva.13245, PMID: 34295367 PMC8288009

[19] Regan T Hori TS Bean TP. A chromosome-scale Mytilus edulis genome assembly for aquaculture, marine ecology, and evolution. G3 Genes|Genomes|Genetics. (2024) 14(8):jkae138. doi: 10.1093/g3journal/jkae138, PMID: 38935082 PMC11304980

[20] Vazzana M Ceraulo M Mauro M Papale E Dioguardi M Mazzola S. Effects of acoustic stimulation on biochemical parameters in the digestive gland of Mediterranean mussel Mytilus galloprovincialis (Lamarck, 1819)a). J Acous Soc Am. (2020) 147:2414–22. doi: 10.1121/10.0001034, PMID: 32359276

[21] Dias PJ Dordor A Tulett D Piertney S Davies IM Snow M. Survey of mussel (Mytilus) species at Scottish shellfish farms. Aquacult Res. (2009) 40:1715–22. doi: 10.1111/j.1365-2109.2009.02274.x

[22] Saco A Rey-Campos M Novoa B Figueras A. Transcriptomic response of mussel gills after a vibrio splendidus infection demonstrates their role in the immune response. Front Immunol. (2020) 11:615580. doi: 10.3389/fimmu.2020.615580, PMID: 33391288 PMC7772429

[23] Wale MA Simpson SD Radford AN. Noise negatively affects foraging and antipredator behaviour in shore crabs. Anim Behav. (2013) 86:111–8. doi: 10.1016/j.anbehav.2013.05.001

[24] Erbe C MacGillivray A Williams R. Mapping cumulative noise from shipping to inform marine spatial planning. J Acoust Soc Am. (2012) 132:El423–8. doi: 10.1121/1.4758779, PMID: 23145705

[25] McKenna MF Wiggins SM Hildebrand JA. Relationship between container ship underwater noise levels and ship design, operational and oceanographic conditions. Sci Rep. (2013) 3:1760. doi: 10.1038/srep01760

[26] Bean TP Farley H Nascimento-Schulze J Regan T. Scottish oyster mortality event and association with Vibrio aestuarianus. Aquacult Rep. (2024) 39:102480. doi: 10.1016/j.aqrep.2024.102480

[27] Travers M-A Barbou A Le Goïc N Huchette S Paillard C Koken M. Construction of a stable GFP-tagged Vibrio harveyi strain for bacterial dynamics analysis of abalone infection. FEMS Microbiol Lett. (2008) 289:34–40. doi: 10.1111/j.1574-6968.2008.01367.x, PMID: 19054091

[28] Stabb EV Ruby EG. RP4-based plasmids for conjugation between Escherichia coli and members of the Vibrionaceae. Methods in Enzymology (2002) 358:413–26. doi: 10.1016/s0076-6879(02)58106-4, PMID: 12474404

[29] Martins E Figueras A Novoa B Santos RS Moreira R Bettencourt R. Comparative study of immune responses in the deep-sea hydrothermal vent mussel Bathymodiolus azoricus and the shallow-water mussel Mytilus galloprovincialis challenged with Vibrio bacteria. Fish Shellfish Immunol. (2014) 40:485–99. doi: 10.1016/j.fsi.2014.07.018, PMID: 25089010

[30] Oliveros JC. Venny. An interactive tool for comparing lists with Venn Diagrams(2007). Available online at: http://bioinfogp.cnb.csic.es/tools/venny/index.html (Accessed July 17, 2025).

[31] Zhong Z Guo Y Zhou L Chen H Lian C Wang H. Transcriptomic responses and evolutionary insights of deep-sea and shallow-water mussels under high hydrostatic pressure condition. Sci Total Environ. (2024) 949:175185. doi: 10.1016/j.scitotenv.2024.175185, PMID: 39089385

[32] Charman M Colbourne TR Pietrangelo A Kreplak L Ridgway ND. Oxysterol-binding protein (OSBP)-related protein 4 (ORP4) is essential for cell proliferation and survival. J Biol Chem. (2014) 289:15705–17. doi: 10.1074/jbc.M114.571216, PMID: 24742681 PMC4140924

[33] Nathalie Tijet CH. René Feyereisen: The cytochrome P450 gene superfamily in Drosophila melanogaster: Annotation, intron-exon organization and phylogeny. Gene. (2001) 262:189–98. doi: 10.1016/S0378-1119(00)00533-3, PMID: 11179683

[34] Sun Y Zhang X Wang G Lin S Zeng X Wang Y. PI3K-AKT signaling pathway is involved in hypoxia/thermal-induced immunosuppression of small abalone Haliotis diversicolor. Fish Shellfish Immunol. (2016) 59:492–508. doi: 10.1016/j.fsi.2016.11.011, PMID: 27825946

[35] Oertle T Huber C van der Putten H Schwab ME. Genomic structure and functional characterisation of the promoters of human and mouse nogo/rtn4. J Mol Biol. (2003) 325:299–323. doi: 10.1016/s0022-2836(02)01179-8, PMID: 12488097

[36] O’Regan L Blot J Fry AM. Mitotic regulation by NIMA-related kinases. Cell Div. (2007) 2:25. doi: 10.1186/1747-1028-2-25, PMID: 17727698 PMC2018689

[37] Yucel SS Lemberg MK. Signal peptide peptidase-type proteases: versatile regulators with functions ranging from limited proteolysis to protein degradation. J Mol Biol. (2020) 432:5063–78. doi: 10.1016/j.jmb.2020.05.014, PMID: 32464132

[38] Balbi T Fabbri R Cortese K Smerilli A Ciacci C Grande C. Interactions between Mytilus galloprovincialis hemocytes and the bivalve pathogens Vibrio aestuarianus 01/032 and Vibrio splendidus LGP32. Fish Shellfish Immunol. (2013) 35:1906–15. doi: 10.1016/j.fsi.2013.09.027, PMID: 24080469

[39] Jiao Y Gu Z Luo S Deng Y. Evolutionary and functional analysis of MyD88 genes in pearl oyster Pinctada fucata martensii. Fish Shellfish Immunol. (2020) 99:322–30. doi: 10.1016/j.fsi.2020.02.018, PMID: 32060010

[40] Toubiana M Gerdol M Rosani U Pallavicini A Venier P Roch P. Toll-like receptors and MyD88 adaptors in Mytilus: complete cds and gene expression levels. Dev Comp Immunol. (2013) 40:158–66. doi: 10.1016/j.dci.2013.02.006, PMID: 23485525

[41] Wang T Sun Y Jin L Thacker P Li S Xu Y. Aj-rel and Aj-p105, two evolutionary conserved NF-kappaB homologues in sea cucumber (Apostichopus japonicus) and their involvement in LPS induced immunity. Fish Shellfish Immunol. (2013) 34:17–22. doi: 10.1016/j.fsi.2012.09.006, PMID: 23022054

[42] Liu W Liu B Zhang G Jia H Zhang Y Cen X. Molecular and functional characterization of a short-type peptidoglycan recognition protein, ct-PGRP-S1 in the giant triton snail charonia tritonis. Int J Mol Sci. (2022) 23. doi: 10.3390/ijms231911062, PMID: 36232364 PMC9570181

[43] Premachandra HKA Elvitigala DAS Whang I Lee J. Identification of a novel molluscan short-type peptidoglycan recognition protein in disk abalone (Haliotis discus discus) involved in host antibacterial defense. Fish Shellfish Immunol. (2014) 39:99–107. doi: 10.1016/j.fsi.2014.04.018, PMID: 24811007

[44] Gorbushin AM. Identification of peptidoglycan recognition proteins in hemocytes and kidney of common periwinkle Littorinalittorea. Fish Shellfish Immunol. (2022) 120:11–4. doi: 10.1016/j.fsi.2021.11.009, PMID: 34774730

[45] Wang X Qu X Lu X Chen M Ning J Liu H. Characterization of TRAF genes and their responses to Vibrio Anguillarum challenge in Argopecten scallops. Fish Shellfish Immunol. (2023) 135:108675. doi: 10.1016/j.fsi.2023.108675, PMID: 36906048

[46] Ren Y Ding D Pan B Bu W. The TLR13-MyD88-NF-kappaB signalling pathway of Cyclina sinensis plays vital roles in innate immune responses. Fish Shellfish Immunol. (2017) 70:720–30. doi: 10.1016/j.fsi.2017.09.060, PMID: 28958897

[47] Qi Z Xu Y Wang X Wang S Zhang Q Wang Z. TLR13, TLR22, TRAF6, and TAK1 in the soiny mullet (Liza haematocheila): Molecular characterization and expression profiling analysis. Dev Comp Immunol. (2020) 112:103774. doi: 10.1016/j.dci.2020.103774, PMID: 32634525

[48] Qi P He Y Liao Z Dong W Xia H. Molecular cloning and functional analysis of tumor necrosis factor receptor-associated factor 6 (TRAF6) in thick shell mussel, Mytilus coruscus. Fish Shellfish Immunol. (2018) 80:631–40. doi: 10.1016/j.fsi.2018.05.053, PMID: 29859313

[49] Li X Wang D Su Z Mao X. TNFAIP3-interacting protein 1 (ABIN-1) negatively regulates caspase-8/FADD-dependent pyroptosis. FEBS J. (2025) 292:1972–90. doi: 10.1111/febs.17404, PMID: 39827378

[50] Luo Y Li C Landis AG Wang G Stoeckel J Peatman E. Transcriptomic profiling of differential responses to drought in two freshwater mussel species, the giant floater Pyganodon grandis and the pondhorn Uniomerus tetralasmus. PLoS One. (2014) 9:e89481. doi: 10.1371/journal.pone.0089481, PMID: 24586812 PMC3934898

[51] Jo P-G Choi Y-K An K-W Choi C-Y. Osmoregulation and mRNA Expression of a Heat Shock Protein 68 and Glucose-regulated Protein 78 in the Pacific oyster Crassostrea gigas in Response to Salinity Changes. J Aquacult. (2007) 20:205–11.

[52] Cellura C Toubiana M Parrinello N Roch P. Specific expression of antimicrobial peptide and HSP70 genes in response to heat-shock and several bacterial challenges in mussels. Fish Shellfish Immunol. (2007) 22:340–50. doi: 10.1016/j.fsi.2006.06.007, PMID: 16926100

[53] Youtsey LS Reece KS Snyder RA McDowell JR. Transcriptomic profile of the northern hard clam (Mercenaria mercenaria) in response to short-term low salinity exposure. Front Mar Sci. (2025) 12:1574486. doi: 10.3389/fmars.2025.1574486

[54] Aleng NA Sung YY MacRae TH Abd Wahid ME. Non-lethal heat shock of the asian green mussel, perna viridis, promotes hsp70 synthesis, induces thermotolerance and protects against vibrio infection. PLoS One. (2015) 10:e0135603. doi: 10.1371/journal.pone.0135603, PMID: 26288319 PMC4546054

[55] Ray Chaudhuri A Nussenzweig A. The multifaceted roles of PARP1 in DNA repair and chromatin remodelling. Nat Rev Mol Cell Biol. (2017) 18:610–21. doi: 10.1038/nrm.2017.53, PMID: 28676700 PMC6591728

[56] Witkop EM Proestou DA Gomez-Chiarri M. The expanded inhibitor of apoptosis gene family in oysters possesses novel domain architectures and may play diverse roles in apoptosis following immune challenge. BMC Genomics. (2022) 23:201. doi: 10.1186/s12864-021-08233-6, PMID: 35279090 PMC8917759

[57] Dong Y Zhang Z Huang Y Tan X Li X Huang M. The role of HMGB2 in the immune response of Nile tilapia (Oreochromis niloticus) to streptococcal infection. Fish Shellfish Immunol. (2024) 153:109845. doi: 10.1016/j.fsi.2024.109845, PMID: 39159774

[58] Vazzana M Celi M Maricchiolo G Genovese L Corrias V Quinci EM. Are mussels able to distinguish underwater sounds? Assessment of the reactions of Mytilus galloprovincialis after exposure to lab-generated acoustic signals. Comp Biochem Physiol Part A: Mol Integr Physiol. (2016) 201:61–70. doi: 10.1016/j.cbpa.2016.06.029, PMID: 27371112

[59] Gerdol M Moreira R Cruz F Gómez-Garrido J Vlasova A Rosani U. Massive gene presence-absence variation shapes an open pan-genome in the Mediterranean mussel. Genome Biol. (2020) 21:275. doi: 10.1186/s13059-020-02180-3, PMID: 33168033 PMC7653742

[60] Saco A Rey-Campos M Gallardo-Escárate C Gerdol M Novoa B Figueras A. Gene presence/absence variation in Mytilus galloprovincialis and its implications in gene expression and adaptation. iScience. (2023) 26:107827. doi: 10.1016/j.isci.2023.107827, PMID: 37744033 PMC10514466

[61] Gualandi N Fracarossi D Riommi D Sollitto M Greco S Mardirossian M. Unveiling the Impact of Gene Presence/Absence Variation in Driving Inter-Individual Sequence Diversity within the CRP-I Gene Family in Mytilus spp. Genes (Basel). (2023) 14. doi: 10.3390/genes14040787, PMID: 37107545 PMC10138031

[62] Gerdol M Saco A Riommi D Greco S Kireta D Edomi P. The mytilin gene cluster: Shedding light on the enigmatic origin of mussel dispensable genes. Fish Shellfish Immunol. (2025) 161:110286. doi: 10.1016/j.fsi.2025.110286, PMID: 40118229

[63] Krone R Gutow L Joschko TJ Schröder A. Epifauna dynamics at an offshore foundation–implications of future wind power farming in the North Sea. Mar Environ Res. (2013) 85:1–12. doi: 10.1016/j.marenvres.2012.12.004, PMID: 23312860

