# Supplementary figures and images for: Anthropogenic noise exposure suppresses the immune response in Mytilus spp. following Vibrio splendidus challenge
Source: Front Immunol. 2025 Sep 18;16:1657667. doi: 10.3389/fimmu.2025.1657667 (PMC12488463; doi:10.3389/fimmu.2025.1657667)

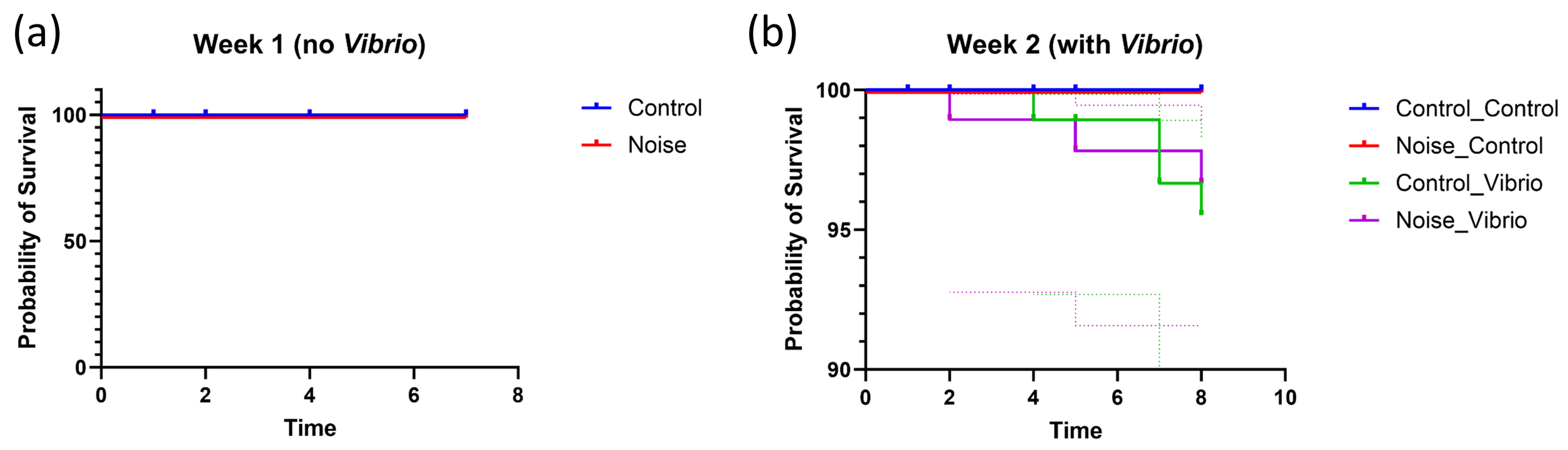

Supplement: Supplementary Figure 1 — Mussel survival during noise exposure and after Vibrio challenge. (a) Survival during the 7-day noise exposure period(n = 208 mussels per tank, 1 tank per group). (b) Survival during the week following Vibrio or mock challenge, across four groups: Control, Noise Only, Vibrio Only, and Noise + Vibrio (n = 101 mussels per tank, 1 tank per group). Dotted lines represent 95% confidence intervals for survival estimates, calculated by GraphPad Prism using the Kaplan-Meier method. No significant differences in mortality were observed (log-rank test, p > 0.05). [file Image1.jpeg]
